# Response: Commentary: False-Positive Effect in the Radin Double-Slit Experiment on Observer Consciousness as Determined With the Advanced Meta-Experimental Protocol

**DOI:** 10.3389/fpsyg.2020.596125

**Published:** 2020-12-03

**Authors:** Jan Walleczek, Nikolaus von Stillfried

**Affiliations:** Phenoscience Laboratories, Berlin, Germany

**Keywords:** metascience reform movement, advanced meta-experimental protocol, confirmatory analysis, HARKing, Radin double-slit experiment

To better prevent false discoveries in science, the metascience reform movement seeks to improve the reliability of the scientific process at multiple levels (e.g., www.metascience.com and www.metascience2019.org). In this context, an advanced meta-experimental protocol (AMP) was developed by Walleczek at Phenoscience Laboratories (www.phenoscience.com) and implemented in an experimental study that was reported by Walleczek and von Stillfried (2019) in this journal.

A recent commentary by Radin et al. (2020), as well as an earlier critique (Radin et al., 2019), heavily misrepresented the methodology and statistical interpretation of this experiment that was commissioned by the funder (www.fetzer-franklin-fund.org) to be performed blindly by Radin. Importantly, data encryption was used in this replication experiment to prevent p-hacking and HARKing, i.e., undisclosed hypothesizing after the results are known (Kerr, 1998). HARKing occurs when a researcher gives the (false) impression that the used form of statistical analysis had been pre-registered, or planned, before unblinding and examining the data, when—in truth—the analysis was developed *post-hoc*. HARKing increases greatly the risk of mistaking a false discovery for a true discovery or vice versa.

Here we provide a summary of the two major ways in which Radin et al. (2019, 2020) deviated from the funder-approved, pre-specified protocol for this commissioned study. For full disclosure and transparency, and given the elaborate construction of the misrepresentation by Radin et al. (2019, 2020), the detailed research record is available in Walleczek and von Stillfried (2020).

First, Radin et al. (2020) claimed that Walleczek and von Stillfried (2019) failed to report a “true-positive outcome” for this experiment. The research record for this funder-commissioned study shows clearly, however, that the used analytic method—a chi-square test—was not pre-specified in advance. That is, regarding the chi-square test analysis, the research record contradicts the recent claim by Radin et al. (2020) that “the analytic methods in the experiment were established before to prevent p-hacking.” Thus, regrettably, this claim by Radin et al. (2020) amounts to an example of the malpractice of HARKing. Briefly, the simple reason why this chi-square test was present in the Matlab-script, which Radin et al. (2020) explained that they had “examined,” is the following: It was a *post-hoc* modified version of the script—sent to the authors by Radin himself—at an earlier point in time, but years after the original pre-specified analysis had already been completed. Crucially, that version of the script contained, in addition to the planned statistical analyses, Radin's *post-hoc* analysis based on the (non-directional) chi-square test. Walleczek and von Stillfried (2019) review (i) the original null results of the non-directional analysis that was actually pre-specified by Radin, and (ii) the test predictions by Radin in the original research contract.

A second major claim by Radin et al. (2020) suggested that the significant false-positive effect reported by Walleczek and von Stillfried (2019) is not really significant because it should be corrected for multiple testing. This suggestion amounts to yet another case of HARKing because Radin et al. (2020) abandoned (i) the planned predictive single-testing strategy and—without disclosing this fact—switched to (ii) an unplanned random multiple-testing strategy—subsequent to breaking the blinding code. Briefly, Radin et al. (2020) misrepresent the pre-specified research design by suggesting—without mentioning the test predictions—that “… the experiment involved eight comparisons performed on non-overlapping data partitioned from a single dataset.” For explanation, Radin et al. (2020) here falsely suggest that the eight different hypotheses merely posited the random occurrence of (arbitrary) effects for “a single dataset”; however, in truth, a different specific prediction was tested with each of eight dedicated (single) data sets (Walleczek and von Stillfried, 2019). Therefore, statistical corrections for non-predictive, i.e., random, multiple testing would thus violate the AMP-based test strategy which implemented the planned outcome predictions as pre-specified by Radin in 2011: True-positive effects were predicted for only two (12.5%) of the 16 possible measurement outcomes of the eight single-test categories. That crucial fact is entirely ignored by the (randomizing) *post-hoc* analysis promoted by Radin et al. (2020) using HARKing. Walleczek and von Stillfried (2019) further clarify why the AMP-based predictive approach—contrary to Radin et al. (2020)—does not entail the standard multiple comparison problem which does not distinguish between random and predictive testing (e.g., Tukey, 1991; Curran-Everett, 2000; Frane, 2015).

Given that the criticisms by Radin et al. (2019, 2020) are based on HARKing, the results reported in Walleczek and von Stillfried (2019) remain valid exactly as reported: (i) a “false-positive effect, which would be indistinguishable from the predicted true-positive effect, was significant at p = 0.021 (σ = −2.02; N = 1,250 test trials)” and (ii) “no statistically significant effects” in those two groups for which true-positives were predicted to occur. These observations are consistent with an independent statistical reanalysis of the Radin double-slit (DS)-experiment by Tremblay (2019) and a replication attempt by Guerrer (2019). Tremblay reported significant false-positives in control groups and Guerrer found significant effects with *post-hoc* analyses only, but null results only with the planned formal analysis: “The formal experiments… were not statistically significant” (Guerrer, 2019).

Given these findings from three independent sources in 2019 (Guerrer, 2019; Tremblay, 2019; Von Stillfried and Walleczek, 2019; Walleczek and von Stillfried, 2019), it is increasingly apparent that the Radin DS-experiment is prone to producing false discoveries, in particular with *post-hoc* analyses, including HARKing. Short of radical improvements in the research standards for the Radin DS-experiment, it is likely that false discoveries will continue to be promulgated, including for replication experiments. Importantly, the probability of a true discovery is increased greatly if empirical proof is available for the absence of false-positive effects in this experiment. However, Radin et al. (2019, 2020) do not report a credible control mechanism that would be capable of distinguishing a (potential) true-positive observer effect from a false-positive observer effect. Therefore, the present authors call for the implementation of advanced control-test strategies, like the confirmatory AMP-based research design (Walleczek and von Stillfried, 2019, 2020), for empirically detecting and preventing uncontrolled false-positive effects in the Radin DS-experiment.

## Author Contributions

JW and NS jointly wrote this commentary and have full agreement regarding its content.

## Conflict of Interest

The authors declare that the research was conducted in the absence of any commercial or financial relationships that could be construed as a potential conflict of interest.

## References

[B1] Curran-Everett D. (2000). Multiple comparisons: philosophies and illustrations. Am. J. Physiol. Regul. Integr. Comp. Physiol. 279, R1–R8. 10.1152/ajpregu.2000.279.1.R1 10896857

[B2] Frane A. V. (2015). Planned hypothesis tests are not necessarily exempt from multiplicity adjustment. J. Res. Pract. 11:P2. Available online at: http://jrp.icaap.org/index.php/jrp/article/view/514/417

[B3] Guerrer G. (2019). Consciousness-Related Interactions in a Double-Slit Optical System. Available online at: https://osf.io/qdkvx

[B4] Kerr N. L. (1998). HARKing: hypothesizing after the results are known. Pers. Soc. Psychol. Rev. 2, 196-217. 10.1207/s15327957pspr0203_4 15647155

[B5] Radin D. Wahbeh H. Michel L. Delorme A. (2019). Psychophysical Effects in Double-slit Interference Patterns: Response to a Critique. Available online at: https://psyarxiv.com/9csgu/

[B6] Radin D. Wahbeh H. Michel L. Delorme A. (2020). Commentary: false-positive effect in the radin double-slit experiment on observer consciousness as determined with the advanced meta-experimental protocol. Front. Psychol. 11:726. 10.3389/fpsyg.2020.00726 32351435 PMC7174750

[B7] Tremblay N. (2019). Independent re-analysis of alleged mind-matter interaction in double-slit experimental data. PLoS ONE 14:e0211511. 10.1371/journal.pone.0211511 30730911 PMC6366771

[B8] Tukey J. W. (1991). The philosophy of multiple comparisons. Stat. Sci. 6, 100–116.

[B9] Von Stillfried N. Walleczek J. (2019). Sham-Experiments Reveal a Statistical Error and the Need for Confirmatory Research in the Radin Double-Slit Experiment. Available online at: https://osf.io/nyq5x/

[B10] Walleczek J. von Stillfried N. (2019). False-positive effect in the Radin double-slit experiment on observer consciousness as determined with the advanced meta-experimental protocol. Front. Psychol. 10:1891. 10.3389/fpsyg.2019.01891 31507481 PMC6714546

[B11] Walleczek J. von Stillfried N. (2020). False-Positive Effect in the Radin Double-slit Experiment: HARKing is used by Radin et al. to Misrepresent the Advanced Meta-experimental Protocol used in Walleczek and von Stillfried (2019). Available online at: https://psyarxiv.com/a2vkn/

